# Serum sCD14, PGLYRP2 and FGA as potential biomarkers for multidrug‐resistant tuberculosis based on data‐independent acquisition and targeted proteomics

**DOI:** 10.1111/jcmm.15796

**Published:** 2020-09-23

**Authors:** Jing Chen, Yu‐Shuai Han, Wen‐Jing Yi, Huai Huang, Zhi‐Bin Li, Li‐Ying Shi, Li‐Liang Wei, Yi Yu, Ting‐Ting Jiang, Ji‐Cheng Li

**Affiliations:** ^1^ Institute of Cell Biology Zhejiang University School of Medicine Hangzhou China; ^2^ Yuebei People’s Hospital Shaoguan China; ^3^ Department of Clinical Laboratory Zhejiang Hospital Hangzhou China; ^4^ Department of Pneumology Shaoxing Municipal Hospital Shaoxing China

**Keywords:** biomarker, data‐independent acquisition, FGA, multidrug‐resistant tuberculosis, parallel reaction monitoring, PGLYRP2, sCD14

## Abstract

Multidrug‐resistant tuberculosis (MDR‐TB), defined as tuberculosis (TB) resistant to at least isoniazid and rifampicin, is a major concern of TB control worldwide. However, the diagnosis of MDR‐TB remains a huge challenge to its prevention and control. To identify new diagnostic methods for MDR‐TB, a mass spectrometry strategy of data‐independent acquisition and parallel reaction monitoring was used to detect and validate differential serum proteins. The bioinformatic analysis showed that the functions of differential serum proteins between the MDR‐TB group and the drug‐sensitive tuberculosis group were significantly correlated to the complement coagulation cascade, surface adhesion and extracellular matrix receptor interaction, suggesting a disorder of coagulation in TB. Here, we identified three potential candidate biomarkers such as sCD14, PGLYRP2 and FGA, and established a diagnostic model using these three candidate biomarkers with a sensitivity of 81.2%, a specificity of 90% and the area under the curve value of 0.934 in receiver operation characteristics curve to diagnose MDR‐TB. Our study has paved the way for a novel method to diagnose MDR‐TB and may contribute to elucidate the mechanisms underlying MDR‐TB.

## 1 INTRODUCTION

Multidrug‐resistant tuberculosis (MDR‐TB) is a subtype of tuberculosis (TB) that is resistant to at least isoniazid and rifampicin. It requires long‐term (18‐24 months) treatment, in which second‐line drugs are less effective and can cause adverse reactions.^1^ Although worldwide the diagnosis and treatment of TB have been improved in recent years, leading to a decreasing trend in the overall incidence of tuberculosis, MDR‐TB is still a major global concern. China is a high MDR‐TB burden country. There are various causes of MDR‐TB infection, for example infection with resistant strains of *Mycobacterium tuberculosis* (MTB) and unfavourable duration of treatment with insufficient medication intake. According to a survey carried out in China for MDR‐TB, 43.8% of the patients who were retreated for MDR‐TB did not complete the primary treatment,^2^ due to the lack of strict implementation of directly observed treatment strategy (DOTS), adverse reactions and high treatment cost.

A multi‐centre study has found that the mortality rate of drug‐sensitive tuberculosis (DS‐TB) was about 6%, while the mortality rate of MDR‐TB was about 57.1%, suggesting the risk of death was greatly increased in MDR‐TB.^3^ Early initiation of TB treatment can reduce mortality by 20%‐35%.^4^

However, the detection of TB is still a challenge. According to the World Health Organization report, in 2017, approximately 56% of TB cases were diagnosed by positive pathogen detection worldwide, and this rate was about 32% in China, due to one of the reasons that a single pathogen test was not applicable to diagnose all TB.^5^ For smear‐negative detection, most (68%) Chinese smear‐negative TB patients were given diagnostic treatment (the treatment is effective) without drug resistance test or bacteriological confirmation, leading to a increased risk of excessive side‐effects, drug resistance and decreased drug efficacy. Furthermore, only approximately 1.6% of Chinese TB patients reported were rifampin‐resistant, leaving more than 80% of cases undetected.^6^, ^7^

There are two main diagnostic methods for MDR‐TB. One is based on culture phenotypes, such as Drug Susceptibility Testing (DST), which is the principal standard for the diagnosis of MDR‐TB. However, the samples have to be cultured for a long period of time (12 weeks).^5^ Another is based on molecular methods, such as XPERT MTB/RIF, which can rapidly detect the resistance to rifampicin. But the detection rate of XPERT MTB/RIF is low (72.5%‐76.9%) in smear‐negative/culture‐positive samples,^8^ and its additional problem is false positivity which may lead to an over‐treatment.^9^ Overall, these diagnostic methods are suitable for sputum‐positive patients, but not for sputum‐negative patients because of poor sensitivity. They are not applicable either to people with rare sputum.^4^ Therefore, there is an urgent need to develop new, sensitive and efficient diagnostic methods for MDR‐TB.

The emergence of proteomics provides a new approach for the diagnosis of clinical diseases. Proteome is diverse and is the material basis of the complex phenotype of the organism. Its dynamic changes and structural function alterations can directly clarify the mechanisms underlying the pathological conditions of the disease. Thus, proteome is the core of life science research in the post‐genome era and has aroused great concern. Previously, the weak cation exchange (WCX) magnetic beads combined with protein chip‐based surface‐enhanced laser desorption/ionization time‐of‐flight mass spectrometry (SELDI‐TOF MS) technique have been used to obtain differential protein peaks in the laboratory, which had difficulties to identify peptides, and was unsuitable for detection of high molecular weight proteins (>100 kD).^10^ With advances in the proteomics technology, data‐dependent acquisition (DDA) and data‐independent acquisition (DIA) strategies have emerged to identify differential peptides in a broader range than SELDI‐TOF MS. DDA tends to select the peptide with the strongest fragment signal. The precursor (MS1) signal is usually less selective than the fragment ion (MS2) signal, resulting in problems of randomness and reproducibility.^11^

Data‐independent acquisition collects data from a single sample, allowing fragmentation and analysis of all peptides in a given m/z window and enabling complete recording and highly reproducible quantification of all MS2 scans,^12^ so it is more suitable for extensive screening, qualitative and quantitative analysis of large batches of samples.

Previously, immunoassay‐based techniques have been applied to validate candidate protein biomarkers after DDA screening,^13^ which are non‐specific and low‐throughput. The development of targeted mass spectrometry technology enables high‐throughput and sensitive protein quantification. For example, parallel reaction monitoring (PRM), the proteomics of monitoring all products of the target peptide, can simultaneously filter out all other peptides and proteins in the sample, to enhance the specificity and sensitivity of the quantification and achieve validation of one or several specific proteins.

Our study is the first to use the technology of DIA combined with PRM to detect differential candidate proteins in the sera of patients with MDR‐TB for identification of potential biomarkers. By analysing the combination of obtained candidate biomarkers, the present study may contribute to establish biological basis for a new approach to the laboratory diagnosis of MDR‐TB.

## 2 MATERIALS AND METHODS

### 2.1 Study design and inclusion criteria

In this study, 80 TB cases (40 MDR‐TB cases and 40 DS‐TB cases) were enrolled between May 2013 and September 2018 from the Shaoxing Municipal Hospital (China) and the First Hospital of Jiaxing (China). In addition, 40 healthy individuals (HC) were collected from the Zhejiang Hospital (China). Age and gender characteristics of the enrolled participants are shown in Table 1. The distributions of age and gender between different groups showed no statistical difference. The research programme was conducted in compliance with the ethical guidelines of the Helsinki Declaration and was approved by the Ethics Committee of Zhejiang University (China). The informed consent was signed by all enrolled participants.

**Table 1:** Age and gender characteristics of included cases

	HC	MDR‐TB	DS‐TB	*p* value
Training set				
Age	33.40 ± 10.59	39.26 ± 15.52	36.55 ± 14.95	.421
Sex (Male)	20 (10)	20 (12)	20 (14)	.435
Validation set				
Age	36.85 ± 9.18	41.55 ± 12.07	35.55 ± 13.57	.245
Sex (Male)	20 (12)	20 (14)	20 (16)	.386
Total				
Age	35.12 ± 9.94	40.43 ± 3.72	36.05 ± 14.11	.146
Sex (Male)	40 (22)	40 (26)	40 (30)	.172

Note

Gender was analysed by chi‐square test, and age was analysed by one‐way ANOVA.

The diagnosis of TB is based on the diagnostic criteria issued by the Ministry of Health of China.^14^ TB patients met one of the following criteria: (a) positive bacteriological examination (two positive sputum smears on microscopy for acid‐fast bacilli, or one positive sputum smear for acid‐fast bacilli with chest imaging revealing evidence of typical TB lesions, or one positive sputum smear for acid‐fast bacilli and one positive sputum culture identified as the MTB complex); (b) two negative sputum smears for acid‐fast bacilli, positive mycobacterial culture confirmed as the MTB complex and typical tuberculosis lesions on chest X‐ray examination; (c) positive molecular biology examination and typical TB lesions on chest X‐ray examination; (d) histopathological changes of TB on the lung lesions tissue samples; and (e) diagnosis as tracheal, bronchial TB or tuberculous pleurisy.

For TB patients, sputum examination (smear or culture), drug sensitivity test and strain identification of the MTB complex were performed. The definite diagnosis of MDR‐TB was made when the enrolled patient had at least two times positive sputum smears, and the patient was resistant to at least two important drugs such as isoniazid and rifampicin, and was proven as MDR‐TB by a bacteriological examination, together with the evidence from the patients’ complete medical history, physical examination and chest X‐ray features. The patients enrolled in the DS‐TB group were confirmed to be sensitive to the drugs by DST. The exclusion criteria included the presence of diabetes, extra‐pulmonary TB, sarcoidosis, hepatitis B, allergic diseases or use of immunosuppressant or immunomodulator drugs within 6 months. The control group comprised healthy individuals with similar distribution of age and gender as the TB cases.

Serum sampling was performed following the Human Proteome Organization (HUPO) recommendations.^15^ Fasting blood samples were collected, centrifuged at 840 *g* for 10 min at 4°C and stored at −80°C.

### 2.2 Liquid phase separation and mass spectrometry

Data‐independent acquisition was used to screen candidate proteins in the training set, and PRM was used in the validation set. The work flow of mass spectrum is shown in Figure S1. The parameters of liquid phase separation and mass spectrometry are listed in Table 2. And the proteomic data were uploaded to the databases of ProteomeXchange Consortium. The samples were pre‐treated (File S1) and chromatographed using a nanolitre flow HPLC system, Easy nLC‐1200. Samples after nanoscale HPLC separation were analysed by Q‐Exactive HF mass spectrometer (Thermo Scientific). High‐abundance proteins were removed to retain low‐abundance proteins by Multiple Affinity Removal Column (Hu‐14) (Agilent Technologies).

**Table 2:** The parameters and materials of liquid phase separation and mass spectrometry

Data collection	DDA mode	DIA mode	PRM qualitative analysis	PRM quantitative analysis
Trap column	EasySprayTM C18 Trap Column (Thermo Scientific, 3 μm, 75 μm*2 cm)	EasySprayTM C18 Trap Column (Thermo Scientific, 3 μm, 75 μm*2 cm)	Home‐made column (100 μm*50 mm, 5 μm‐C18)	Home‐made column (100 μm*50 mm, 5 μm‐C18)
Analytical column	EasySprayTM C18 LC chromatographic column (Thermo Scientific, 2 μm, 75 μm*50 cm)	EasySprayTM C18 LC chromatographic column (Thermo Scientific, 2 μm, 75 μm*50 cm)	Home‐made tip‐column (75 μm*200 mm, 3 μm‐C18)	Home‐made tip‐column (75 μm*200 mm, 3 μm‐C18)
Flow velocity	250 nL/min	250 nL/min	300 nL/min	5 μL/min
Liquid phase separation gradient	0‐97 min, linear gradient of B liquid was from 8% to 30%; 97‐110 min, linear gradient of B liquid was from 30% to 100%; 110‐120 min, linear gradient of B liquid rose to 100% and was maintained.	0‐97 min, linear gradient of B liquid was from 10% to 30%; 97‐110 min, linear gradient of B liquid was from 30% to 100%; 110‐120 min, the linear gradient of B liquid rose to 100% and was maintained.	0‐2 min, linear gradient of B liquid was from 5% to 10%, 2‐45 min, linear gradient of liquid B was from 10% to 30%; 45‐55 min, the linear gradient of liquid B was from 30% to 100%; 55‐60 min, the linear gradient of liquid B was maintained at 100%.	0‐2 min, linear gradient of B liquid was from 5% to 10%, 2‐45 min, linear gradient of liquid B was from 10% to 30%; 45‐55 min, the linear gradient of liquid B was from 30% to 100%; 55‐60 min, the linear gradient of liquid B was maintained at 100%.
MS analysis duration	120 min	120 min	60 min	60 min
MS detection mode	Positive ions	Positive ions	Positive ions	Positive ions
MS1 parameters				
MS1 scanning range	300‐1800 m/z	350‐1650 m/z	300‐1800 m/z	300‐1800 m/z
Mass spectrometry resolution	60 000 (@m/z 200)	120 000 (@m/z 200)	60 000 (@m/z 200)	60 000 (@m/z 200)
AGC target	3e6	3e6	3e6	3e6
Maximum IT	200 ms	50 ms	50 ms	200 ms
MS2 parameters				
MS2	20 MS2 scans	DIA data acquisition mode, 30 windows	Targeted shotgun scanning mode, 20 MS2 scans	20 MS2 scans
Isolation window	1.6 Th	1.6 Th	1.6 Th	1.6 Th
Mass spectrometry resolution	30 000 (@m/z 200)	30 000 (@m/z 200)	15 000(@m/z 200)	30 000 (@m/z 200)
AGC target	3e6	3e6	1e5	3e6
Maximum IT	120 ms	auto	50 ms	120 ms
MS2 Activation Type	HCD	HCD	HCD	HCD
Normalized collision energy	27	30	27	27
Spectral data type	/	Profile	/	/
Software parameters				
Maxquant_1.5.3.17	Enzyme: trypsin; max miss cleavage site: 2; fixed modification: Carbamidomethyl(C); dynamic modification: Oxidation(M) and Acetyl (Protein N‐term)			
Skyline	Enzyme: trypsin/P; missed cleavage site: 0			
Spectronaut Pulsar X_12.0.20491.4	Retention time prediction type: dynamic iRT; interference on MS2 level correction: enabled; cross run normalization: enabled			

Note

Chromatographic separation was performed using HPLC system Easy nLC‐1200 (Thermo Scientific), and mass spectrometry was performed using Q‐Exactive HF (Thermo Scientific). For liquid phase separation, buffer A was 0.1% aqueous solution of formic acid, and solution B was 0.1% aqueous solution of acetonitrile and formic acid (acetonitrile of 84%). Maxquant was used of database retrieval. Skyline was used of analysis of PRM, and Spectronaut was used of DIA data processing.

Abbreviations: DDA, data‐dependent acquisition; DIA, data‐independent acquisition; PRM, parallel reaction monitoring

For DIA analysis, 2 μg of peptide was taken from each sample, and the iRT standard peptide was spiked according to the volume ratio of the sample: iRT of 3:1. The DDA method was used to build libraries, and the DIA mode was conducted for qualitative and quantitative analysis to obtain differential proteins. MaxQuant 1.5.3.17 software was used for database search, and the library was constructed by Spectronaut pulsar X 12.0.20491.4 software. The iRT peptide sequence was added into the database (>Biognosys|iRTKit|Sequence_fusionLGGNEQVTRYILAGVENSKGTFIIDPGGVIRGTFIIDPAAVIRGAGSSEPVTGLDAKTPVISGGPYEYRVEATFGVDESNAKTPVITGAPYEYRDGLDAASYYAPVRADVTPADFSEWSKLFLQFGAQGSPFLK). And database was downloaded from Uniprot (human_156639_20170105. fasta, human_159691_20170829. fasta, *Mycobacterium tuberculosis*_136378_ 20190617. fasta). DIA data were compared by secondary mass spectrometry. The protein identified had to pass the set cut‐off of false discovery rate (FDR) < 1%.

Parallel reaction monitoring was applied to validate the identified differential proteins. After pre‐treatment, 2 μg of peptides was taken from each sample, and 20 fmol of standard peptide (PRTC: ELGQSGVDTYLQTK) was incorporated for detection. Peptide scores of the identification >40 were required to ensure reliable results (File S2). According to the results of qualitative analysis, the identified target peptides were screened, the trusted peptides were retained, and the trusted peptides suitable for PRM analysis were introduced into the mass spectrometry software Xcalibur for PRM method setting. After three times of PRM tests, the data of the PRM original file were analysed by Skyline 3.7.0 software, and the target proteins and the target peptides were quantified.

### 2.3 Bioinformatic analysis

Blast2GO was applied to perform Gene Ontology (GO) annotation for the target protein set, which could be summarized as a sequential process: sequence alignment (Blast) and GO Entry Extraction (Mapping, GO Annotation, and InterProScan Supplemental Notes [Annotation Augmentation]). The Kyoto Encyclopedia of Genes and Genomes (KEGG) pathway annotation was performed for the target protein set by KAAS (KEGG Automatic Annotation Server) software. In order to analyse the target protein clusters, the quantitative information was firstly normalized (normalized to (−1, 1) interval). Then, the two‐dimensional abundance of samples and proteins (distance algorithm: Euclidean, Connection linkage) was constructed by Complexheatmap R package (R Version 3.4) and a hierarchical clustering heatmap was generated. The application software SIMCA‐P 14.1 (Umetrics, Umea, Sweden) was used for pattern recognition. After the data were pre‐processed by Paretoscaling, PCA multidimensional statistical analysis was performed. The correlation and interaction network was drawn by Cytoscape 3.6.1 software.

### 2.4 Statistical analysis

The Fisher's exact test was conducted to compare the distribution of GO categories or KEGG pathways between the target protein set and the overall protein set, and to perform enrichment analysis on the GO categories or KEGG pathways of the target protein set. The composition ratio was analysed by the chi‐square test, and the parameter data were subjected to a *t* test or analysis of variance. The scatter diagrams were generated by Graphpad Prism 5 software, and the ROC curve analysis was conducted in Medcalc software. Significant correlation analysis was defined as r > 0.4 or r < −0.4 using a two‐tailed *p* value (*P* < .01) by spearman analysis.

## 3 RESULTS

### 3.1 Differential protein candidates of DIA

**Figure 1:**
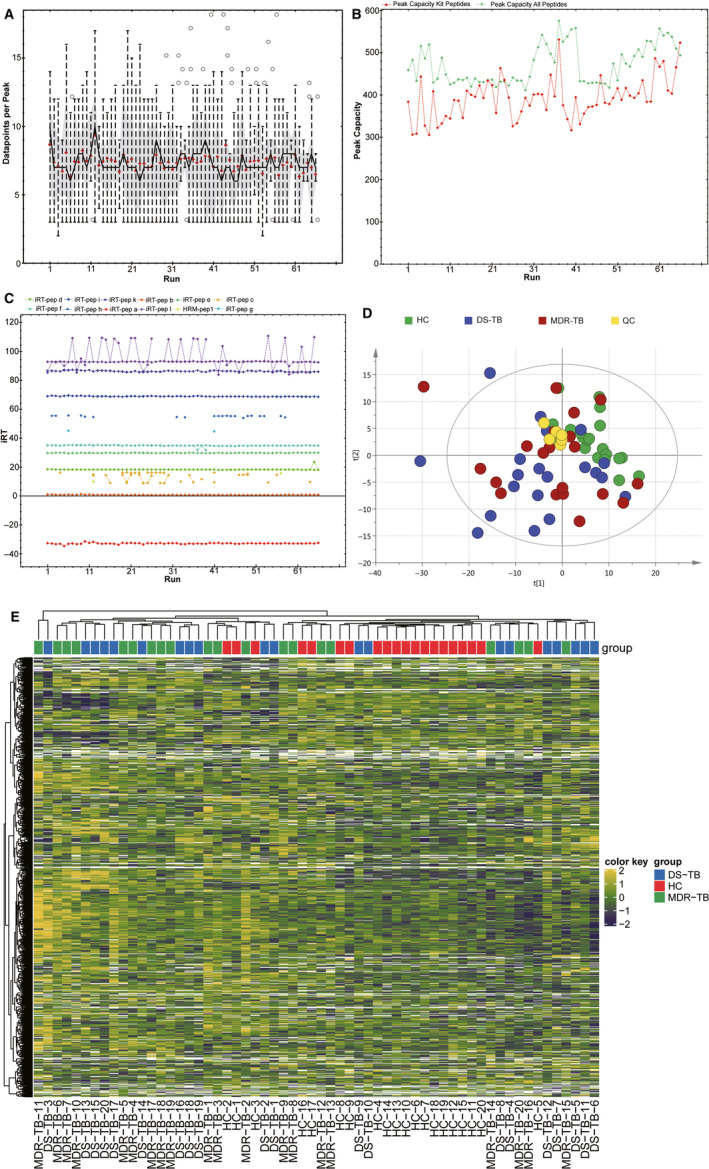
Quality control and quantitative heat map of DIA. A, Average data points per peak: the average data points per peak were 7.2, which met the requirements of quantitative analysis. B, Column peak capacity statistics: the abscissa was the order of the samples, the green line was the data of all peptides, and the red one was the data of the iRT internal standard. Peak capacity represented the separation and analysis capability of the column. The average peak capacity was 471, indicating better separation and analysis. C, Chart of iRT elution time: the main iRTs were detected and the retention time was generally stable. D, Chart of principal component analysis (PCA): quality control (QC) was evaluated using the coefficient of variation CV and PCA analysis. E, Quantitative heatmap of DIA

**Figure 2:**
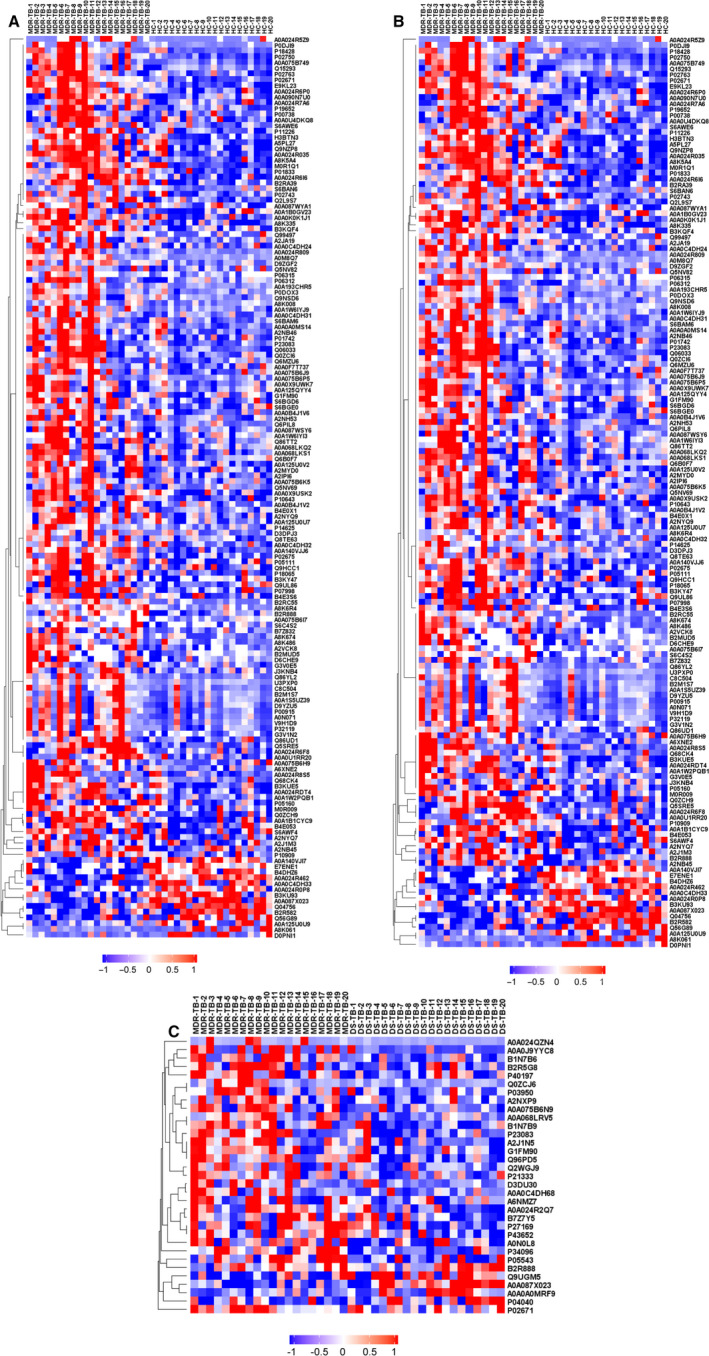
Differential protein clusters. A, MDR‐TB/HC. B, DS‐TB/HC. C, MDR‐TB/DS‐TB

The differential proteins in the MDR‐TB group were screened and quantified by DIA (Figure 1E). The quality control of the data is shown in Figure 1A‐D. The FDR method was applied to conduct multiple testing correction. *Q* Value 0.01 was set as the threshold which was equivalent to FDR 0.01. A total of 1000 proteins were identified, and 813 proteins were present in more than 50% of the samples. The differential clusters were generated with proteins presenting a fold change of more than 1.2 or less than 0.7 and a *P* < .05 (Figure 2). A total of 157 differential proteins (143 up‐regulated and 14 down‐regulated) were identified between the HC group and the MDR‐TB group, and a total of 33 differential proteins (28 up‐regulated and 5 down‐regulated) were identified between the MDR‐TB group and the DS‐TB group. In the comparison between the DS‐TB group and the HC group, 170 differential proteins (135 up‐regulated and 35 down‐regulated) were discovered.

**Figure 3:**
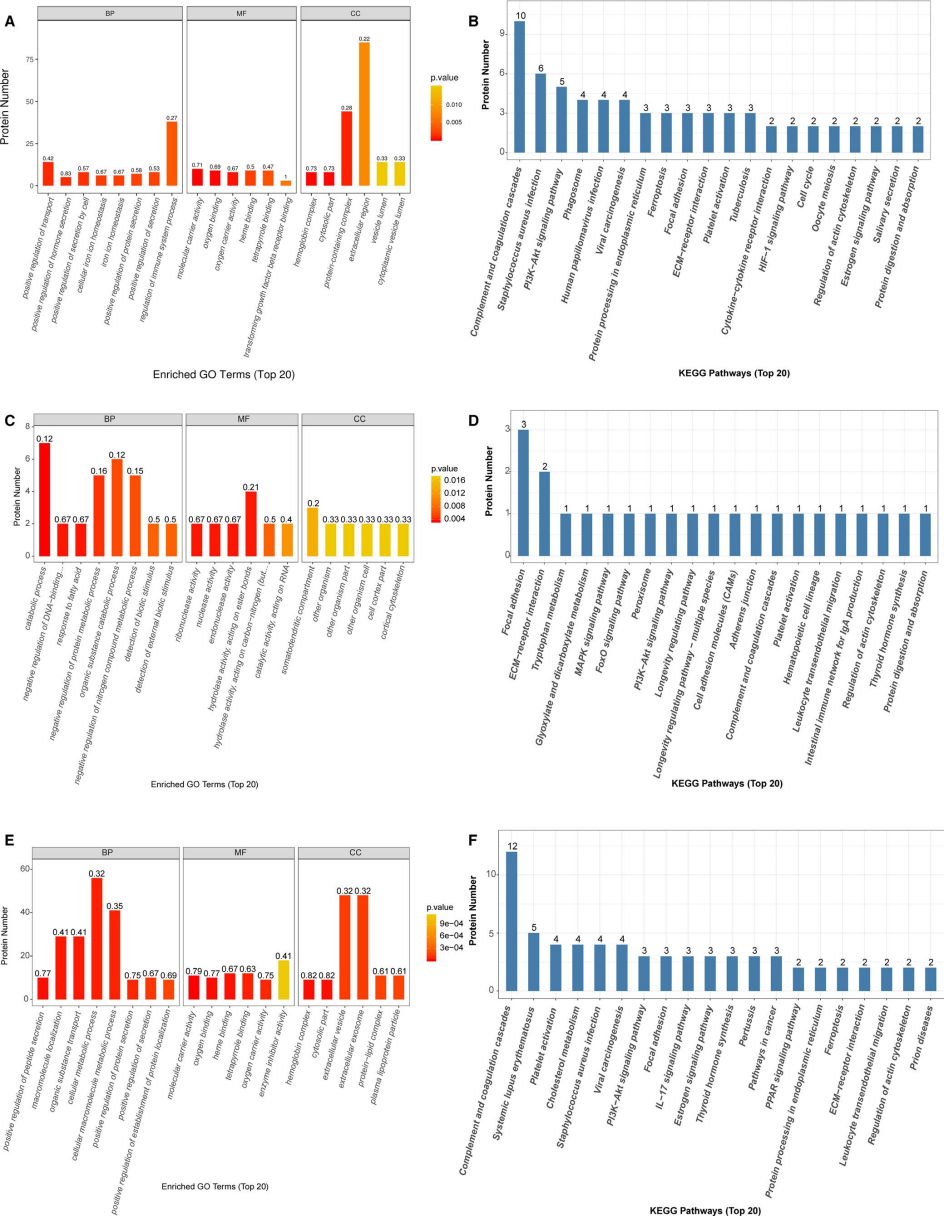
GO and KEGG analysis. A and B, MDR‐TB/HC. C and D, MDR‐TB/DS‐TB. E and F, DS‐TB/HC

The GO and KEGG functional enrichment analyses were performed on the differential proteins (Figure 3). The KEGG analysis showed that the differential proteins in the MDR‐TB group were mainly related to the complement coagulation cascade, compared with the HC group. The biological process of GO suggested the differential proteins in the MDR‐TB group played an important role in the transduction pathway of immune response‐regulating signalling and response‐activating signal transduction. The binding of cofactor and the cellular region was the most significant molecular function and cellular component enriched in the MDR‐TB group. In addition, the KEGG analysis suggested that the differential proteins between the MDR‐TB group and the DS‐TB group were involved in surface adhesion and ECM receptor interaction. The GO analysis indicated that these proteins were enriched in hyperoxia and enzyme activities and located mainly on the membrane, in the extracellular regions and cellular regions. Compared with the HC group, differential proteins in the DS‐TB group were significantly associated with the complement coagulation cascade, the binding of cofactors and immune responses, such as interleukin (IL)‐1 production and regulation, and Toll‐like receptor (TLR) signalling pathways.

### 3.2 Parallel reaction monitoring

**Figure 4:**
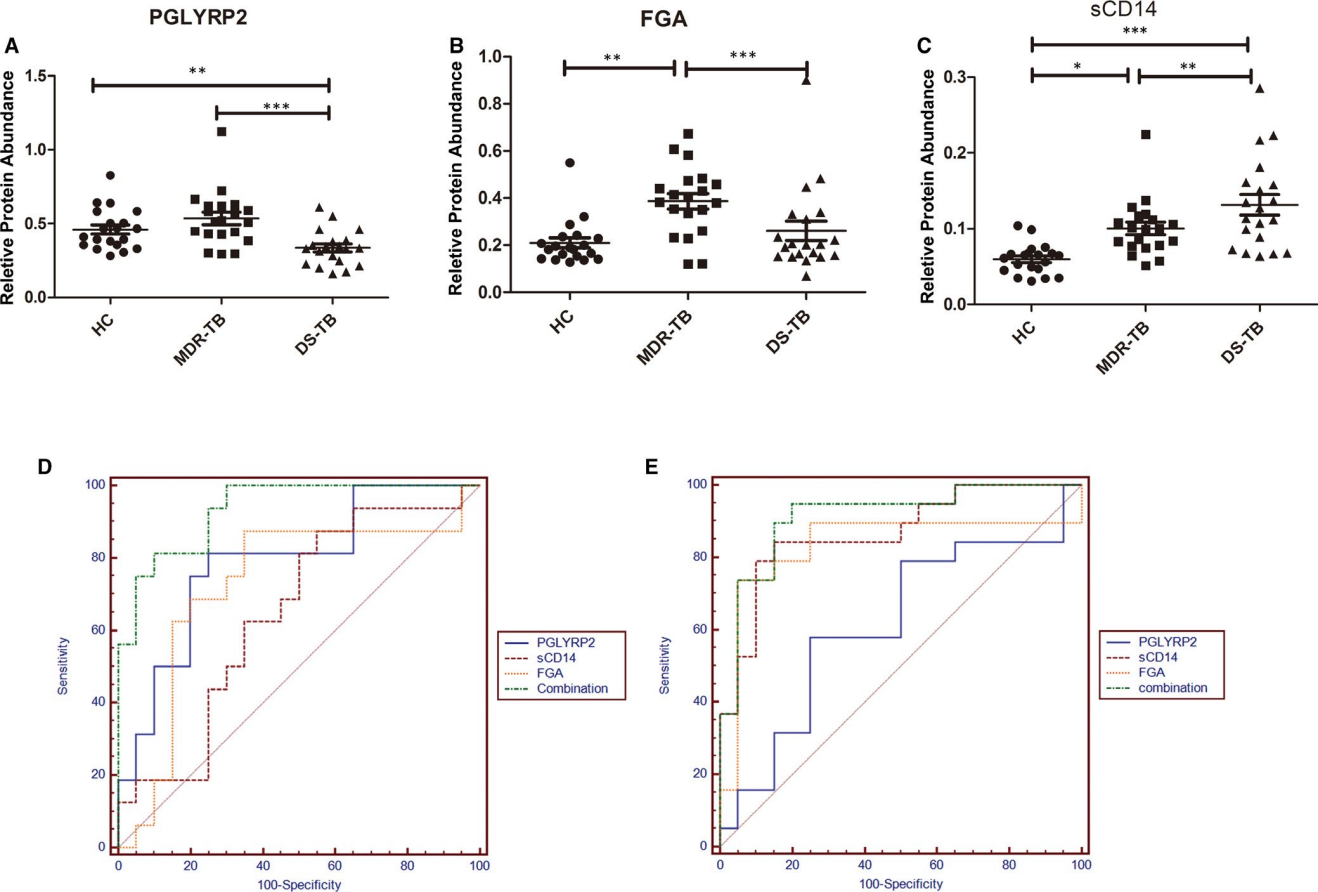
Relative protein abundance and ROC analysis. The ordinate was the peak area. A, Relative protein abundance of PGLYRP2. B, Relative protein abundance of FGA. C, Relative protein abundance of sCD14. D, ROC analysis of MDR‐TB/DS‐TB. E, ROC analysis of MDR‐TB/HC

According to the results from the protein qualitative analysis, the identified target peptides were screened to retain the trusted peptides. After the targeted monitoring of differential proteins in mixed samples, mass spectrometry experiments showed that 18 target proteins could be accurately identified. Compared with the DS‐TB group, the abundances of peptidoglycan recognition protein 2 (PGLYRP2, Swissprot: Q96PD5), fibrinogen alpha chain (FGA, Swissprot: P02671) and monocyte differentiation antigen CD14 (sCD14, Swissprot: B2R888) were significantly up‐ (*P* < .001), up‐ (*P* < .001) and down‐regulated (*P* < .01), respectively, in the MDR‐TB group (Figure 4A‐C).

### 3.3 ROC analysis

To assess the sensitivity and specificity of these three proteins in the diagnosis of MDR‐TB, we performed the multivariate logistic regression analysis and the ROC curve analysis. The sensitivity of PGLYRP2 was 80%, the specificity was 80%, and the area under the curve (AUC) value was 0.827 to distinguish between the MDR‐TB group and the DS‐TB group. The sensitivity and specificity of FGA for the detection of MDR‐TB were 90% and 65%, respectively, with AUC value of 0.765. Similarly, the sensitivity and specificity of sCD14 were 85% and 50%, with AUC value of 0.655. The three proteins were combined to establish a diagnostic model with a sensitivity and specificity of 81.2% and 90%, respectively, with AUC value of 0.934, which was higher than the single protein model (Figure 4D). To distinguish between the MDR‐TB group and the HC group, PGLYRP2, FGA and sCD14 obtained sensitivities and specificities of 60% and 75%, 75% and 95%, and 80% and 90%, respectively. The combination of these three proteins was established a diagnostic model with a sensitivity of 94.7%, a specificity of 80%, and the AUC value of 0.913, which was much higher than the models established by single proteins, such as PGLYRP2 (0.627), FGA (0.838) and sCD14 (0.875) (Figure 4E).

### 3.4 Network of interaction and correlation

**Figure 5:**
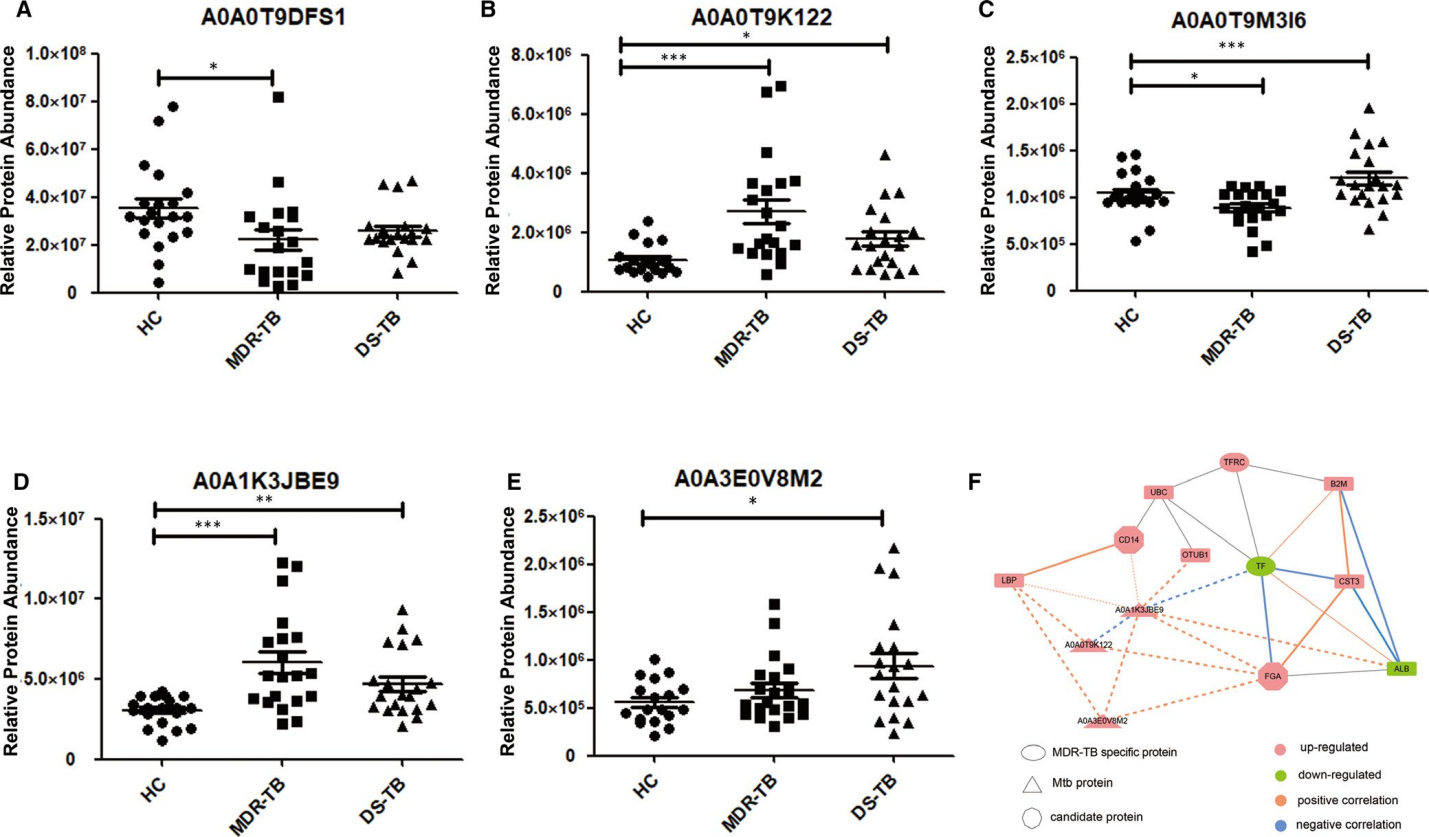
Differential proteins of MTB complex and interaction and correlation analysis. A, Fatty acid CoA ligase. B, Putative integral membrane protein. C, Histidine‐t RNA ligase. D, Restriction endonuclease S subunit. E, FAD‐dependent monooxygenase (Fragment). ***P < .001, **P < .01 and *P < .05. F, Interaction and correlation analysis of differential proteins of TB patients and MTB complex. The solid lines showed the interaction of differential proteins of TB patients, and the imaginary lines represented the correlation analysis of differential proteins of TB patients and MTB complex. The thickness of the line indicated the strength of the correlation. Significant correlation: *r* > .4 or *r* < −.4 and*P* < .01

We also detected differential proteins secreted by the MTB complex in the sera of TB patients (Figure 5A‐E). A network of above candidate biomarker proteins and differential MTB proteins was established (Figure 5F). Lipopolysaccharide‐binding protein (LBP), sCD14, ubiquitin C‐like protein (UBC), ubiquitin thioesterase protein (OTUB1), transferrin receptor (TFRC), beta‐2‐microglobulin (B2M), cystatin (CST3) and FGA were up‐regulated in the MDR‐TB group and the DS‐TB group, compared with the HC group. However, transferrin (TF) and albumin (ALB) were down‐regulated in both TB groups. Correlation of differential MTB proteins and host proteins (LBP, sCD14, OTUB1, TF, FGA and ALB) reflected that the MTB metabolism of MTB complex could change the immune and inflammatory effects of the host. Candidate proteins and MDR‐TB‐specific proteins suggested immunity‐ and inflammation‐related protein changes in the progression of TB.

## 4 DISCUSSION

In this study, we used a new mass spectrometry strategy to identify potential biomarkers for MDR‐TB diagnosis. Compared with the HC group, the sCD14 abundance was significantly up‐regulated in the DS‐TB group and the MDR‐TB group, but the sCD14 abundance in the MDR‐TB group was lower than that in the DS‐TB group. Studies have reported that the sCD14 may serve as a potential biomarker for TB,^16^ but there is no study available regarding the role of sCD14 in the progression of TB. The sCD14 protein is an indicator of monocyte activation.^17^ In the initial stage of TB infection, monocytes rapidly migrate to the site of infection and differentiate into macrophages, thereby causing host defence responses; thus, the sCD14 abundance was increased in TB. Decreased sCD14 abundance in the MDR‐TB group may indicate a decrease in monocyte activation, compared with the DS‐TB group. The increased level of sCD14 can also be observed in other infections or lung diseases, but is usually lower than that of TB.^18^, ^19^

PGLYRP2 is an *N*‐acetylmuramyl‐l‐alanine amidase that hydrolyses bacterial peptidoglycan. It plays a scavenger role and hydrolyses the link between residues of L‐amino acid and N‐acetylcytokines in glycopeptides of the cell wall. PGLYRP2 has a pro‐inflammatory effect and synergizes with TLR2 and TLR4 to promote macrophage activation.^20^, ^21^ It has been demonstrated that PGLYRP2 can regulate the recruitment of neutrophils after *S pneumoniae* infection, suggesting a host protection role of PGLYRP2.^22^ Our results indicated that PGLYRP2 abundance was significantly up‐regulated in the MDR‐TB group, compared with the DS‐TB group, which may be related to an enhancement of the body's defence response.

Fibrinogen is a member of the plasminogen (Plg)‐fibrinolytic system, which can bind to the surface of microorganisms by the Plg receptor, and can be activated by a host activator to produce proteolytic enzyme plasmin (Plm). Studies have found that the MTB complex interacts with the plasminogen system to convert microbes into proteolytic organisms, thereby enhancing their invasive potential.^23^ We have previously found that the level of fibrinogen in TB was significantly increased compared to that in non‐TB controls,^10^ revealing significant abnormalities of coagulation in TB. Our results indicated that FGA abundance was significantly up‐regulated in the MDR‐TB group, suggesting that the activation of the fibrinolytic system was closely related to the progression of TB. Although in the present study no statistically significant difference was observed in the FGA level between the DS‐TB group and the HC group, the DS‐TB group showed an upward trend, which was consistent with the previous findings reported by Wang et al^24^ In sputum and saliva, a previous work has shown no statistically significant difference in FGA levels was observed between the latent TB group and the HC group, but a significant difference between the active TB group and the HC group.^25^ In this study, FGA showed an upward trend in TB groups, indicating that FGA was possibly more up‐regulated in the saliva and sputum than serum. Therefore, we believed that FGA may determine the severity of TB progression.

Interestingly, we also detected a few MTB complex‐secreted proteins in the sera of TB patients. Histidine‐tRNA synthetase, a differential protein between the MDR‐TB group and the DS‐TB group, is an aminoacyl‐tRNA synthetase featured by a folded barrel‐like active site comprising an α‐helix and anti‐parallel β‐surrounded by a ring structure. This structure serves as a template to bind the corresponding amino acid and ATP and forms a key component of bacterial protein synthesis.^26^ In addition, aminoacyl‐tRNA synthetase is a potential clinical drug target,^27^, ^28^ suggesting that the secretome and metabolome of the MTB complex may contribute to the pathogenicity and virulence of the strain.

Based on the interaction and correlation analysis, we discovered the possible pro‐inflammatory effects of MTB proteins on the body and weakened host's protective immune response against MTB. There was a positive correlation between the protein abundance of sCD14 and LBP, which has been observed in chronic inflammation patients as well.^29^ The sCD14 abundance also showed a low‐positive correlation to restriction endonuclease S subunit. Compared with the DS‐TB group, the abundance of restriction endonuclease S subunit tended to be up‐regulated in the MDR‐TB group, indicating that the virulent MTB complex could strongly resist the host immune response during infection. We speculated that sCD14 can regulate the recognition of bacterial proteins by interacting with UBC and LBP. A variety of bacterial proteins have shown a strong correlation with FGA, which may be the main influencing factor of host coagulation abnormalities, and can be further verified by functional studies. It has been demonstrated that the MTB complex did not increase TF’s abundance in the lungs but in the macrophages of granulomatous lung lesions.^30^ In the MDR‐TB group, the level of TF showed a specific down‐regulation, which may explain the weakened host protective immune response against drug‐resistant MTB.

In this study, we used DIA combined with PRM to identify potential biomarkers for MDR‐TB. Previously, the shotgun proteomics technique, the most widely used and standardized discovery strategy based on DDA, has been used to screen candidate biomarkers in diseases. Through digesting the protein into peptides in vitro and performing tandem mass spectrometry, the subsequently obtained mass spectrometry data are aligned to a protein sequence database or a spectral library to search for protein identification. Although the method offers a wide coverage, the disadvantages are the lack of high sensitivity, specificity, reproducibility, and low overlap rate (35%‐60%) in repeated experiments and more missing values.^31^ In DIA, the data passe through the independent acquisition mode of the sequential window. All ionized compounds in a given sample fall within a specified mass range, broken in a systematic and unbiased manner, which overcomes the shortcomings of the shotgun proteomics’ semi‐randomity. When injecting the same sample and using the same mass spectrometer under the same conditions, DIA is superior to DDA in detecting peptides and related proteins, and has a high measuring reproducibility.^32^, ^33^ Therefore, DIA is more suitable for the screening of a wide range of biomarkers.

However, in terms of quantitative sensitivity, the peptide quantification sensitivity of DIA is 3‐10 times lower than traditional targeted proteomics (SRM/PRM).^34^ Therefore, we performed a targeted validation for the candidate proteins from DIA screening. Technically, the detection of targeted proteomics is more accurate to monitor low‐abundance proteins and peptides, and can be targeted for qualitative and quantitative analyses of single or multiple target proteins. It can replace the previous non‐specific and low‐throughput techniques based on immunoassay, like enzyme‐linked immunosorbent assay and Western blot, and can achieve higher resolution and mass accuracy. However, appropriate methods for specific proteins are still necessary.^35^ PRM can simultaneously monitor the proteomics of entire products of each target peptides, and can filter out all other peptides and proteins in the sample with fewer parameters that need to be optimized, which are beneficial to a shorter detection time.^36^ However, due to the labour and costs, the total number of the proteins that can be targeted and validated is limited. Hence, PRM is more suitable to analyse the candidate biomarkers in a specific disease.

## 5 CONCLUSION

In summary, we used DIA combined with targeted validation of PRM and obtained three potential protein biomarkers (sCD14, FGA and PGLYRP2) for combinatorial modelling analysis. In the MDR‐TB and DS‐TB groups, we found that the sensitivity and specificity of the ROC curve were 81.2% and 90%, and the AUC value was 0.934, which was higher than the single protein models. Our results laid the foundation to develop the new methods for MDR‐TB diagnosis and may contribute to elucidate underlying mechanisms of MDR‐TB. Our study also illustrated the appropriate application of proteomics in the field of disease biomarkers and may be helpful in the future research of TB pathology‐related functions. We believe that the future application of proteomics in the field of biomarkers will be extended and will help us better understand the mechanisms underlying the disease.

## CONFLICT OF INTEREST

The authors declare that there are no conflicts of interests.

## AUTHOR CONTRIBUTION

**Jing Chen:** Data curation (equal); Formal analysis (lead); Investigation (lead); Methodology (equal); Visualization (equal); Writing‐original draft (lead); Writing‐review & editing (equal). **Yushuai Han:** Data curation (supporting); Formal analysis (equal); Investigation (equal); Methodology (equal); Validation (equal). **Wen‐Jing Yi:** Software (equal). **Huai Huang:** Software (equal). **Zhi‐Bin Li:** Validation (lead). **Liying Shi:** Investigation (equal). **Liliang Wei:** Investigation (equal). **Yi Yu:** Software (supporting); Validation (supporting). **Tingting Jiang:** Funding acquisition (equal); Supervision (equal). **Ji‐Cheng Li:** Conceptualization (equal); Funding acquisition (equal); Methodology (equal); Resources (equal); Writing‐review & editing (equal).

## SUPPORTING INFORMATION

The mass spectrometry proteomics data have been deposited to the ProteomeXchange Consortium (http://proteomecentral.proteomexchange.org) via the iProX partner repository^37^ (dataset identifier of DIA data: PXD019856) and PRIDE database (dataset identifier of PRM data: PXD019604).

## Supporting information

Figure S1

File S1

File S2

## Data Availability

The data that support the findings of this study are available from the corresponding author upon reasonable request.
