# Supplementary figures and images for: Serum sCD14, PGLYRP2 and FGA as potential biomarkers for multidrug‐resistant tuberculosis based on data‐independent acquisition and targeted proteomics
Source: J Cell Mol Med. 2020 Sep 23;24(21):12537–49. doi: 10.1111/jcmm.15796 (PMC7686995; doi:10.1111/jcmm.15796)

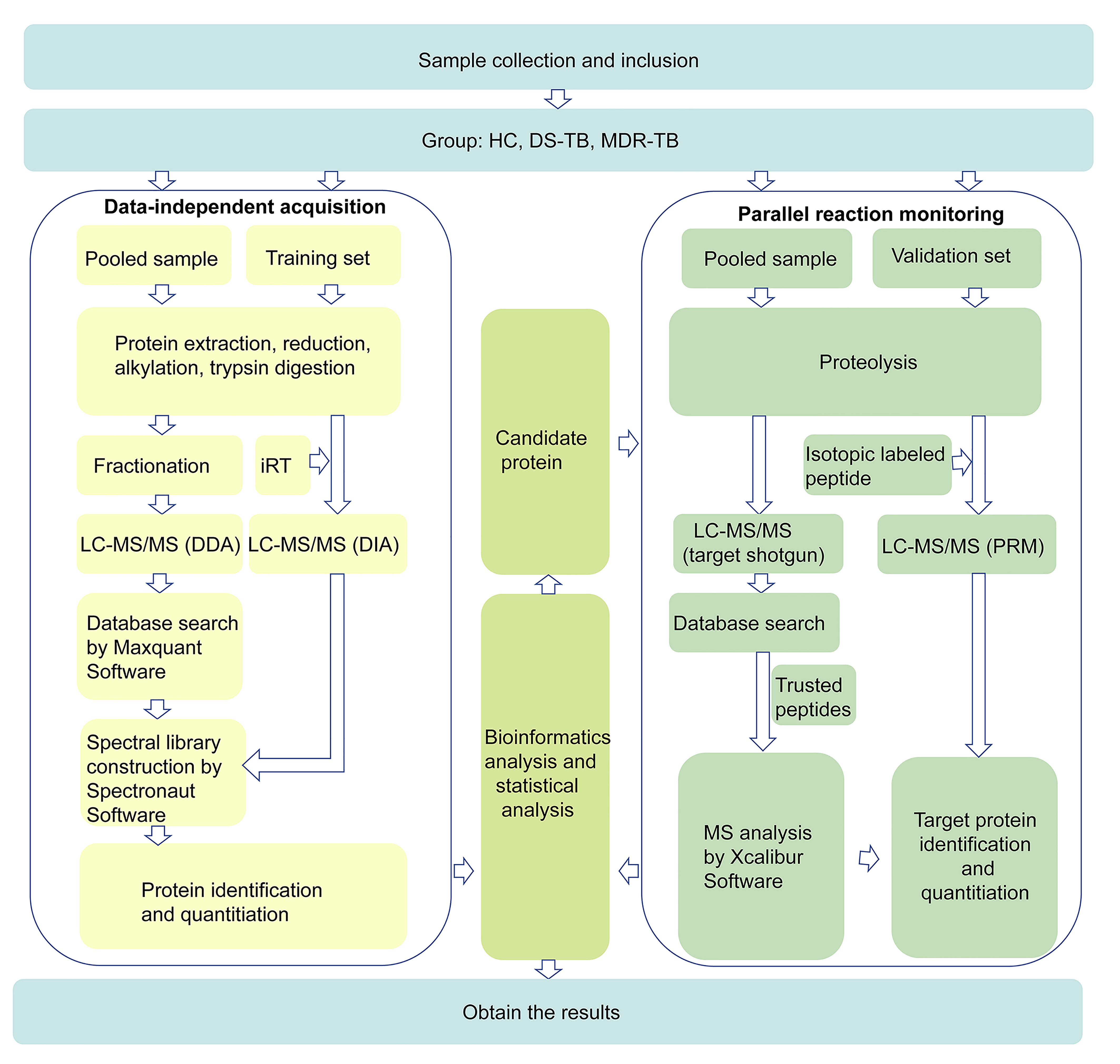

Supplement: Supplementary file 1 — Figure S1 [file JCMM-24-12537-s001.tif]
