# Supplementary material for: Serum sCD14, PGLYRP2 and FGA as potential biomarkers for multidrug‐resistant tuberculosis based on data‐independent acquisition and targeted proteomics
Source: J Cell Mol Med. 2020 Sep 23;24(21):12537–49. doi: 10.1111/jcmm.15796 (PMC7686995; doi:10.1111/jcmm.15796)
Supplement: Supplementary file 2 — File S1 [file JCMM-24-12537-s002.doc]

***DIA analysis***

- ***Protein extraction***

Three samples were taken from each group, and 20 μg of each protein was used as the input for SDS-PAGE electrophoresis to evaluate the consistency between the samples. Trypsin was used to filter-aided sample preparation (FASP) of enzymatic hydrolysis. Dithiothreitol (DTT) was added to a final concentration of 10 mM, placed in a boiling water bath for 15 min, then cooled to room temperature, and mixed with 200 μL of UA buffer (8 M Urea, 150 mM Tris-HCl, pH 8.0). The mixture was transferred to a 10 KDa ultrafiltration tube and centrifuged for 14,000 g for 30 min. Afterwards 200 μl of UA buffer was added and centrifuged for 30 min, and then the filtrate was discarded. 100 μL of IAA (50 mM lodoacetamide in UA) was added to the mixture, shaken at 600 rpm for 1 min, protected from light at room temperature for 30 min, and then centrifuged for 14000 g for 20 min. Next 100 μL of UA buffer was added to the solution, followed by a centrifugation at 14000 g for 20 min and repeated for 3 times. Afterwards, 100 μL of NH4HCO3 buffer (50 mM) was added and centrifuged 2 times at 4000 g, for 20 min. 40 μL of NH4HCO3 buffer (2 μg Lys-C) was added, shaken at 600 rpm for 1 min, and incubated at 37 °C for 4 h, then 2 μg of Trypsin was added to the sample and the sample was left at 37°C for 16 h for the reaction. The sample was placed in a new collection tube and centrifuged at 14000 g, for 15 min. The mixture was centrifuged with 40 μL of NH4HCO3 buffer (50 mM) for 30 min, and the filtrate was collected. OD280 was used to determine the peptide concentration. A total of 100 μg of pool mixed peptides was taken and fractionated by High pH Reversed-Phase Spin Column (Thermo), and 10 fractions were collected. After each component peptide was lyophilized, it was reconstituted with 10 μl of 0.1% FA (formic acid), and the peptide concentration was determined by OD280. Then, 2 μg of the peptide was taken out separately, and the iRT standard peptide was mixed according to the volume ratio of sample:iRT=3:1, and subjected to DDA mass spectrometry with the mass spectrometry time of 2 hours for each component.

- ***Peptide enzymatic hydrolysis***

Each sample was combined with the QC sample and hydrolyzed with Lys-C + Trypsin in the protein solution. The sample was denatured by adding urea lysate (8M urea) and then an appropriate amount of 1M DTT to a final concentration of 20 mM, and restored at 37°C for 60 min. Then 1 M IAA to a final concentration of 50 mM was added and incubated at room temperature for 30 min in the dark. Finally, the urea concentration of the sample was diluted to less than 2M. After enzymatic hydrolysis of Lys-C at a mass ratio of 1:50 (enzyme: protein) for 2 hours, the urea concentration was diluted below 1 M. Trypsin was added at a mass ratio of 1:50 (enzyme: protein) into the enzyme solution and left at 37°C overnight. The samples were desalted, concentrated in vacuo, dried, and the peptides were reconstituted with 10 μl of 0.1% FA, and the peptide concentrations were determined by OD280. Then, 2 μg of each peptide was taken out separately, and the iRT standard peptide was mixed according to the volume ratio sample:iRT of 3:1, and the DIA mass spectrometry was performed with the mass spectrometry time of 2 hours for each component.

***PRM verification***

- ***Sample preparation***

Five samples of an equal amount were randomly chosen from each group of samples and mixed together to make a sample pool, and then enzymatic hydrolysis was conducted directly in the solution.

- ***Protein extraction and pretreatment***

From each sample about 200 μg of protein was taken and then DTT was added to the final concentration of 100 mM. The protein was incubated in boiling water for 15 min, cooled to room temperature. The enzymatic hydrolysis process was carried out by referring to the above-mentioned protein extraction for DIA samples. The enzyme addition ratio of NH4HCO3 buffer (including Trypsin) was 1:50, next centrifuged at 600 rpm for 1 min, then left 37°C for 16 h, and the other steps were the same as ones described in the section of DIA analysis. The digested peptides were desalted and lyophilized, then reconstituted with 0.1% FA, and the peptide concentration was determined by OD280. The peptide information suitable for PRM analysis was imported into the software Xcalibur to set up the PRM method. 2 μg of peptide was taken from each sample, and 20 fmol of standard peptide (PRTC: ELGQSGVDTYLQTK) was incorporated for detection.
